# Dataset for density/temperature correlation of tetramethylammonium hydroxide solutions of various concentrations

**DOI:** 10.1016/j.dib.2022.108679

**Published:** 2022-10-20

**Authors:** Josef Diebold, Yasmin Weiß, Stefan Zürcher, Magnus S. Schmidt

**Affiliations:** a Institute of Precision Medicine, Organic and Bioorganic Chemistry Labs, Medical and Life Sciences Faculty, Furtwangen University, Jakob-Kienzle-Str. 17, D-78054 VS-Schwenningen, Germany; b Team Leader Process Engineering & Laboratory, AP&S International GmbH, Obere Wiesen 9, D-78166 Donaueschingen, Aasen

**Keywords:** Tetramethylammonium hydroxide, Density, Temperature, Archimedes' principle

## Abstract

Densities for various Tetramethylammonium hydroxide (TMAH) solutions at different temperatures have been determined based on Archimedes’ principle using a hydrostatic balance and a vitreous body with a defined volume. Due to the toxicity of the compound, these data are important especially in the field of wet processing techniques for the automated dosage of TMAH.

**Specifications Table**

**Table:** 

Subject	Chemical Engineering: Process Chemistry and Technology
Specific subject area	Tetramethylammonium hydroxide: Correlation between density and temperature for various concentrations has been determined by applying Archimedes’ principle [1,2]. Each measurement has been performed three times allowing the determination of a standard deviation (SD). For each measurement a new set of solutions has been prepared.
Type of data	Table
How the data were acquired	Tempering of the probes was done with a water filled memmert thermostat type W200. Only for the runs at 15°C a MaxQ4000 thermostat from Thermo Scientific was used in order to chill the probes below room temperature. Temperature measurement of solutions was performed by a calibrated thermometer against ice/water and boiling water. Concentrations were adjusted by dilution of a concentrated solution and confirmed by acidimetric titration against 0.5M HCl. Weighing was done on a Mettler PM2000 balance applying equipment allowing hydrostatic weighing according to Archimedes’ principle using a vitreous body with a calibrated volume of 10.005 ml. In order to calibrate the volume of the vitreous body we used Millipore water at 27°C to determine the weight difference according to Archimedes’ principle as 9.97 g. The density of water at 27°C is given as 0,9965 g/ml (Handbook of Chemistry and Physics, 6-11, 72nd edition, 1991-1992). Therefore the volume is calculated to 10.005 ml.
Data format	Raw Analyzed
Description of data collection	A set of Erlenmeyer flasks with each concentration stated in Table 1 were prepared by dilution of a known, concentrated solution. These concentrations have been confirmed by acidimetric titration against 0.5M HCl. For each set a flask was equipped with a thermometer plug to ensure exact temperature measurement in the solution rather than just measure bath temperature. Once reached desired temperature the flasks were transferred one after the other to a hydrostatic balance (Mettler PM2000) to determine the density by Archimedes’ principle. The procedure of weighing was performed quickly and took only half a minute each. Therefore we can assume that the temperature kept stable for this short period of time. Weighing all flasks of a run took only a few minutes and no change of temperature was observed during that time in the thermostat. The whole procedure (diluting, confirming by titration, tempering and weighing) was repeated three times in order to create a data set where calculation of standard deviation was applicable. The standard deviation was calculated in an Excel-File using STABWNA-Formula, which means $\sqrt{\frac{\sum {\left(x-\bar{x}\right)}^{2}}{n}}$
Data source location	Institute of Precision Medicine Organic and Bioorganic Chemistry Labs Faculty Medical and Life Sciences Hochschule Furtwangen | Furtwangen University Jakob-Kienzle-Str. 17, 78054 Villingen-Schwenningen Germany
Data accessibility	1. With the article: [3] 2. Repository name: Mendeley Data Data identification number: DOI: 10.17632/gd8hj92rc6.3 Direct URL to data: https://data.mendeley.com/datasets/gd8hj92rc6/3

## Value of the Data

• Data for the temperature/density/concentration correlation do not exist.
• These data are important especially in wet processing applications i.e. for the calibration of concentration measuring units and are therefore important for industry and science.
• Chemical engineers will benefit from these data especially for the optimization of wet processing applications.

## 1 Objective

Tetramethylammonium hydroxide (TMAH, CAS No. 75-59-2) is a strong basic compound that finds application in both scientific and industrial areas. For example, it is used as a reagent in the thermochemolysis of various biopolymers like lignin [4] and cutan [5] or of biomolecules like carbohydrates [6]. Another example is the anisotropic etching of silicon, which is a key technique in the production of micromechanical devices and therefore is widely used especially in the wet processing industry [7]. Due to its acute toxicity in case of contamination [8,9] implementation of automated concentration monitoring is an important task in the development of new wet processing plants. The measuring unit uses the density of the TMAH solution to check the concentration and therefore needs to be programmed and calibrated with the corresponding information for density/temperature/concentration dependency.

## 2 Data Description

Table 1 shows the correlation between density and temperature for various concentrations of TMAH. As mentioned above the measurements have been performed three times allowing the determination of a standard deviation (SD), which is specified with the results [3]. A clear tendency to higher SD values at higher temperatures and lower concentrations can be observed which correlates to the increasing difference to room temperature and the resulting faster potential loss of temperature during the density measurement itself. Nonetheless, the experiments resulted in a clear dataset for the density/temperature/concentration correlation that can be used for corresponding applications in wet processing technologies.

**Table 1:** Density of various TMAH solutions at various temperatures.

	Density in g/ml at various temperatures								

Conc. in wt%	15°C	25°C	35°C	50°C	70°C	75°C	80°C	85°C	90°C
25	1,0198 ± 0.0005	1.0152 ± 0.0011	1,0115 ± 0.0000	1.0028 ± 0.0012	0.9928 ± 0.0005	0.9898 ± 0.0005	0.9875 ± 0.0008	0.9845 ± 0.0008	0.9822 ± 0.0009
20	1,0138 ± 0.0005	1.0108 ± 0.0000	1,0062 ± 0.0005	1.0008 ± 0.0005	0.9908 ± 0.0012	0.9878 ± 0.0005	0.9845 ± 0.0000	0.9825 ± 0.0008	0.9792 ± 0.0012
17.5	1,0115 ± 0.0000	1.0071 ± 0.0000	1,0045 ± 0.0000	0.9992 ± 0.0005	0.9892 ± 0.0009	0.9858 ± 0.0012	0.9822 ± 0.0005	0.9802 ± 0.0009	0.9768 ± 0.0005
15	1,0095 ± 0.0000	1.0052 ± 0.0000	1,0025 ± 0.0000	0.9972 ± 0.0005	0.9878 ± 0.0012	0.9832 ± 0.0005	0.9808 ± 0.0005	0.9788 ± 0.0012	0.9738 ± 0.0012
12.5	1,0068 ± 0.0005	1.0034 ± 0.0000	1,0008 ± 0.0005	0.9952 ± 0.0009	0.9848 ± 0.0005	0.9818 ± 0.0009	0.9792 ± 0.0009	0.9768 ± 0.0005	0.9722 ± 0.0017
10	1,0052 ± 0.0005	1.0014 ± 0.0000	0,9988 ± 0.0005	0.9932 ± 0.0009	0.9835 ± 0.0008	0.9805 ± 0.0014	0.9775 ± 0.0008	0.9748 ± 0.0005	0.9705 ± 0.0022
8.33	1,0038 ± 0.0005	1.0001 ± 0.0000	0,9985 ± 0.0000	0.9915 ± 0.0014	0.9822 ± 0.0012	0.9785 ± 0.0014	0.9758 ± 0.0009	0.9728 ±0.0005	0.9695 ± 0.0022
5	1,0015 ± 0.0000	0.9991 ± 0.0000	0,9955 ± 0.0000	0.9898 ± 0.0012	0.9795 ± 0.0008	0.9768 ± 0.0017	0.9728 ± 0.0012	0.9705 ±0.0008	0.9682 ± 0.0017
1	0,9982 ± 0.0005	0.9973 ± 0.0000	0,9945 ± 0.0000	0.9875 ± 0.0022	0.9775 ± 0.0016	0.9745 ± 0.0014	0.9708 ± 0.0012	0.9688 ± 0.0012	0.9662 ± 0.0009

## 3 Experimental Design, Materials and Methods

Chemicals and reagents were purchased from Carl Roth or Merck and were used without further purification. The dilution series was prepared using deionized water and graduated flasks. The concentrations have been confirmed by titration against 0.5 M HCl on test basis.

For density measurements the solutions have been transferred to Erlenmeyer flasks which were then equipped with a thermometer plug for direct temperature measurements in the solutions. These then have been placed in a water filled memmert thermostat type W200 and heated to the corresponding temperatures. Only for the runs at 15°C a MaxQ4000 thermostat from Thermo Scientific was used in order to chill the probes below room temperature. The density has been determined for each solution at each temperature three times by using a hydrostatic balance (Mettler PM2000) based on the Archimedes’ principle using a vitreous body with a calibrated volume of 10.005 ml.

## Ethics Statements

No experiments on human participants or animals were carried out during the implementation of these experiments, nor were any human or animal rights violated.

## CRediT Author Statement

**Josef Diebold:** Data curation, Writing – original draft preparation. **Yasmin Weiß:** Investigation, Validation; **Stefan Zürcher:** Resources, Methodology; **Magnus S. Schmidt:** Conceptualization, Supervision, Writing – review & editing.

## Declaration of Competing Interest

The authors declare that they have no known competing financial interests or personal relationships that could have appeared to influence the work reported in this paper.

## Data Availability

TMAH density/concentration/temperature correlation (Original data) (Mendeley Data).
